# Energy Concentration and Phosphorus Digestibility in Hatchery Byproducts Fed to Nursery Pigs

**DOI:** 10.3390/ani9050255

**Published:** 2019-05-21

**Authors:** Jung Yeol Sung, Sang Yun Ji, Ah Reum Son, Beob Gyun Kim

**Affiliations:** 1 Department of Animal Science and Technology, Konkuk University, Seoul 05029, Korea; marcusaura@hanmail.net (J.Y.S.); ardongja@naver.com (A.R.S.); 2 Animal Nutritional Physiology Team, National Institute of Animal Science, Rural Development Administration, Wanju 55363, Korea; syjee@korea.kr

**Keywords:** digestibility, energy, hatchery byproduct, phosphorus, swine

## Abstract

**Simple Summary:**

There is limited information on nutritional value of hatchery byproducts as a swine feed ingredient. The objective of this study was to determine energy concentrations and phosphorus digestibility of hatchery byproducts fed to nursery pigs. Test ingredients were infertile eggs, unhatched eggs, culled chicks, and a mixture of the three. Culled chicks had the greatest energy concentrations, whereas infertile eggs had the lowest values. The standardized total tract digestibility of phosphorus in infertile eggs was greater than that in unhatched eggs, culled chicks, and the mixture. Based on the current results, feed formulation would be possible when using hatchery byproducts in swine diets.

**Abstract:**

The objective was to measure energy concentrations and standardized total tract digestibility (STTD) of phosphorus (P) in hatchery byproducts. In Experiment 1, 20 nursery barrows were used to measure energy concentrations in hatchery byproducts. A basal diet based on corn and dried whey and four additional diets containing 25% of infertile eggs, unhatched eggs, culled chicks, or a mixture of the three hatchery byproducts were prepared. In Experiment 2, the STTD of P was measured using 20 nursery barrows. Four diets containing 25% of the same hatchery byproducts used in Experiment 1 as the sole source of P were prepared, and a P-free diet was prepared to measure basal endogenous losses of P. The marker-to-marker method was employed for total collection. Metabolizable energy in culled chicks was the greatest (4560 kcal/kg as-is basis; *p* < 0.05), whereas infertile eggs had the lowest value (2645 kcal/kg as-is basis; *p* < 0.05). The STTD of P in infertile eggs (81.7%) was greater than that in unhatched eggs, culled chicks, and the mixture (61.6, 53.9, and 47.4%, respectively; *p* < 0.05). In conclusion, culled chicks had the greatest metabolizable energy and infertile eggs had the greatest phosphorus digestibility among the test ingredients.

## 1. Introduction

Weaning is one of the most challenging phases throughout the pig’s life. Replacing milk with solid feeds is stressful for nursery pigs and may cause poor digestion resulting in retarded growth [1]. To maximize the growth performance of nursery pigs, highly digestible animal proteins such as fish meal and spray-dried plasma protein are conventionally included in nursery diets [2,3]. However, these ingredients may increase feed costs because of their high price [4]. Thus, alternative animal protein ingredients can save pig production costs.

Hatchery byproducts (i.e., wastes from hatchery facilities) are defined as “a mixture of egg shells, infertile eggs, unhatched eggs, and culled chicks that have been cooked, dried, and ground, with or without removal of part of the fat” in AAFCO [5]. These byproducts are regarded as potential alternative protein sources for swine diets [6]. Hatchery byproducts are distinguished from poultry byproducts. However, in some cases, hatchery products are incorporated into poultry byproducts [7]. Currently, these products are often disposed of in landfills, resulting in environmental pollution and disposal costs.

An accurate nutritional evaluation of feed ingredients is required for precise feed formulation [8,9]. To the best of our knowledge, however, information on energy and phosphorus (P) utilization of hatchery byproducts by nursery pigs is currently unavailable. Therefore, the objective of this study was to determine digestible energy (DE), metabolizable energy (ME), and standardized total tract digestibility (STTD) of P in hatchery byproducts fed to nursery pigs.

## 2. Materials and Methods

All protocols used in the study were approved by the Animal Care and Use Committee of Konkuk University (approval number: KU17049). Two digestibility experiments were conducted in an environmentally controlled room at Konkuk University.

### 2.1. Preparation of Hatchery Byproducts

Infertile eggs, unhatched eggs, and culled chicks were obtained from a layer hatchery facility (Join Inc., Pyeongtaek, Republic of Korea). Each ingredient was ground and then dried at 130 °C for 20 h in a dryer (DN 2300, Dongnam Tech Inc., Hwaseong, Republic of Korea). The drying temperature and duration were based on preliminary tests to achieve sufficient storability of the ingredients. Infertile eggs and unhatched eggs included egg shells. After the drying process, a mixture of the three ingredients was prepared considering the natural occurrence rate of these ingredients in the hatchery facility and moisture contents (20% dried infertile eggs, 20% dried unhatched eggs, and 60% dried culled chicks) [10].

### 2.2. Animals, Diets, and Experimental Design

Ten barrows with an initial body weight (BW) of 9.4 ± 1.2 kg in Block 1 of Experiment 1 were used to determine energy concentrations in hatchery byproducts. A basal diet based on corn and dried whey was prepared, and four additional diets were prepared by mixing 75% of the basal diet with 25% of test ingredients, respectively (Table 1). The ratio of corn and dried whey was the same in all experimental diets. These five experimental diets were fed to 10 pigs employing a replicated 5 × 3 incomplete Latin square design with 10 pigs, 5 diets, and 3 periods, resulting in 6 replicates per treatment [11]. Two additional replications for each diet were obtained in Block 2 of Experiment 1 using 10 barrows (14.2 ± 1.1 kg initial BW) fed the same experimental diets as in Block 1 of Experiment 1. The experimental design used in Block 2 was a randomized complete block design.

In Block 1 of Experiment 2, 10 barrows (11.6 ± 2.1 kg initial BW) were used to determine the STTD of P in hatchery byproducts. Four experimental diets containing 25% of the hatchery byproducts as the sole source of P were prepared, and a P-free diet was additionally prepared to measure basal endogenous losses of P (Table 2). In Block 2 of Experiment 2, two additional replications of each diet were used with 10 barrows (12.5 ± 0.5 kg initial BW) fed the same experimental diets as in Block 1 of Experiment 2. The experimental design of Experiment 2 was the same as that of Experiment 1.

### 2.3. Feeding and Sample Collection

All pigs were individually housed in metabolism crates equipped with a feeder and a nipple drinker. In all experiments, the quantity of feed provided daily to each pig was calculated as 2.7 times the estimated energy requirement for maintenance (i.e., 197 kcal of ME per kg BW^0.60^) [12] and divided into three equal meals at 8:00, 14:00, and 20:00 h. Each period consisted of a 5-day adaptation period and a 5-day collection period. During collection periods, a total collection method was used to collect feces with the marker-to-marker procedure [13]. Chromium oxide was used as an indigestible marker for initiation and termination of fecal collection. During the collection period, refused feeds were also collected to calculate the amount of actual feed intake. Urine was totally collected from 14:00 h on day 6 to 14:00 h on day 11 in urine buckets over 100 mL of 6 normal hydrochloric acid in Experiments 1 and 2.

### 2.4. Chemical Analyses

Dry matter (DM) [14] and crude protein (CP; method 990.03) [15] in ingredients and diets were determined. Additionally, ash (method 942.05), calcium (Ca; method 935.13), and ether extract (EE; method 920.39) in the ingredients were analyzed [15]. Phosphorus in test ingredients and diets in Experiment 2 was also analyzed (method 946.06) [15]. Concentrations of gross energy (GE) in ingredients and diets were determined using a bomb calorimetry (Parr 1261, Parr Instruments Co., Moline, IL, USA). Feces and urine collected in Experiment 1 were analyzed for GE, while feces collected in Experiment 2 were analyzed for DM and P.

### 2.5. Calculations and Statistical Analyses

In Experiment 1, the difference procedure was used to calculate energy concentrations in test ingredients [16]. In Experiment 2, the apparent total tract digestibility (ATTD) of P was calculated following procedures described by Kwon et al. [17]. The STTD of P was calculated by correcting the ATTD for basal endogenous losses of P derived from the P-free diet.

Data were analyzed using the MIXED procedure of SAS (SAS Inst. Inc., Cary, NC, USA). Data from 2 blocks within each experiment employing the same dietary treatment were pooled for statistical analysis. Dietary treatments were considered as a fixed variable, while block, replication within block, animal within block, and period within block were considered as random variables. The least square means were calculated for each treatment, and differences between least squares means were tested using the PDIFF option with Tukey’s adjustment. The experimental unit was the pig, and an alpha level of 0.05 was used to determine statistical significance.

## 3. Results

Crude protein and GE contents in hatchery byproducts ranged from 32.2 to 67.5% and 4206 to 5884 kcal/kg on an as-is basis, respectively (Table 3). Infertile eggs (31.9%) and unhatched eggs (35.6%) had greater ash contents than culled chicks (8.7%).

In Experiment 1, two pigs fed the mixture diet had diarrhea during the collection period and these two observations were excluded from the data. The energy digestibility of the diet containing infertile eggs was lower (*p* < 0.05) than that of other experimental diets (Table 4). The diet containing culled chicks had the greatest DE and ME among all the experimental diets, whereas DE and ME of the diet containing infertile eggs were the lowest (*p* < 0.05). The DE in the mixture diet was greater (*p* < 0.05) than that in the unhatched egg diet.

The energy values of ingredients showed similar patterns as those in experimental diets (Table 5). Culled chicks had the greatest DE and ME, whereas infertile eggs had the lowest energy values among the test ingredients (*p* < 0.05). The DE in the mixture was greater (*p* < 0.05) than that in unhatched eggs. However, ME concentrations did not differ between unhatched eggs and the mixture. Infertile eggs had the lowest DE:GE and ME:GE ratios among the three ingredients and their mixture (*p* < 0.05). The ME:DE ratio was greater in unhatched eggs compared with that in culled chicks and the mixture (*p* < 0.05).

In Experiment 2, three observations were excluded due to diarrhea and three additional observations were excluded due to feed refusal. Total feed intake and DM intake did not differ between the experimental diets, but the P intake of pigs fed culled chicks and the mixture diet was greater than that of pigs fed infertile eggs and unhatched eggs (*p* < 0.05; Table 6). Total dry feces output was not different between treatments, while the P concentration in feces and P output were greater in pigs fed culled chicks and the mixture compared with those fed infertile eggs (*p* < 0.05). No difference was observed in ATTD of DM. However, the ATTD and STTD of P in infertile eggs was the greatest (*p* < 0.05) among all hatchery byproducts. The ATTD and STTD of P did not differ between unhatched eggs, culled chicks, and the mixture. 

## 4. Discussion

Hatchery byproducts are routinely discarded from hatchery facilities. A total of 6335 tons were discarded in 2015 in the Republic of Korea [18]. Hatchery byproducts are potential substitutes for animal protein sources in swine diets, particularly during the nursery period [6]. To the best of our knowledge, however, nutritional information on hatchery byproducts as a swine feed ingredient is very limited. In the present work, the energy concentrations and P digestibility of hatchery byproducts as feed ingredients for nursery pigs were determined.

In hatchery facilities, hatchery byproducts are pooled and then discarded together. To mimic a natural product in the layer facility where we obtained the ingredients, a mixture of the three ingredients (20% dried infertile eggs, 20% dried unhatched eggs, and 60% dried culled chicks) was prepared in this study. 

Values of CP, EE, and ash in unhatched eggs and culled chicks agreed with previously reported values [19]. Dhaliwal et al. [6] summarized chemical compositions of hatchery byproducts reported in various studies. However, because various types of hatchery byproducts were pooled into a mixture and the inclusion rate of each ingredient was not declared in previous reports, the comparison of chemical components in each ingredient in the current study with reported values is difficult. When comparing the mixture in the present study with reported values, the chemical components of the mixture were within the ranges given in previous studies [6,19,20,21].

The ATTD of GE was not different between the experimental diets except that the infertile egg diet had less GE digestibility than other diets. However, DE in diets differed between the experimental diets mainly due to differences in diet GE contents. Considering that GE concentrations in infertile eggs and unhatched eggs were comparable, the lower DE in infertile eggs compared with that in unhatched eggs was most likely attributed to the lower DE:GE ratio. The low energy digestibility of unhatched eggs may be associated with the drying temperature and duration. The reason for the low energy digestibility of infertile eggs is currently unclear. On day 8 post-fertilization, eggs identified as infertile by inspection were separated from the incubator. The microbial analysis of infertile eggs used in the present study showed no evidence of spoilage [18]. In contrast to the present pig study, interestingly, true ME concentration and energy metabolizability in infertile eggs were found to be greater than those in unhatched eggs when fed to 50-day-old chickens [22]. Further research is warranted to identify the reason for the low energy digestibility of infertile eggs fed to pigs and to search for a method to improve energy digestibility. 

Urinary GE output was not different between the unhatched egg diet and the mixture diet, although the value in the unhatched egg diet was numerically lower than that in the mixture diet (56 vs. 71 kcal/d). Due to this numerical difference in urinary GE output, the ME:DE ratio was greater in the mixture than in unhatched eggs, resulting in no difference in ME between unhatched eggs and the mixture, even though DE was greater in the mixture. This is because urinary GE output is the major factor determining the proportion of DE converted to ME [12]. The DE and ME of the mixture were very close to the values calculated based on the inclusion rate of infertile eggs, unhatched eggs, and culled chicks in the mixture (4203 and 3990 kcal/kg as is, respectively). This indicates that energy values in the hatchery byproducts are additive. 

In the P digestibility trial, the ATTD of DM was expected to be lower in the diet containing infertile eggs and unhatched eggs, as the greater ash concentration in egg shells would have a lower digestibility compared with other components of DM. In the ATTD of DM, however, no difference was found between experimental diets. The lower ATTD and STTD of P in unhatched eggs compared with infertile eggs are likely due to indigestible parts in unhatched eggs including feathers, beaks, and claws, which are mainly composed of keratins [23]. Interestingly, the energy digestibility of unhatched eggs was not largely compromised in Experiment 1 in spite of these indigestible parts. Perhaps this result may have been partially due to a large quantity of ash in the indigestible parts of unhatched eggs. A wider Ca to P ratio and an excessively high Ca concentration in diets have been reported to negatively affect P digestibility and growth performance in pigs, particularly when the P concentration in diets is below the requirement [24,25]. The Ca to P ratio in the diets containing infertile eggs, unhatched eggs, culled chicks, and the mixture was 15.2, 14.4, 2.0, and 4.4, respectively. Except for the culled chick diet, all experimental diets had extremely high Ca to P ratios. However, ATTD and STTD of P in the culled chick diet did not differ from those in the unhatched egg diet or the mixture diet. The reason for this unexpected result is unclear.

## 5. Conclusions

The present work provides values for energy and phosphorus utilization of hatchery byproducts by nursery pigs. Energy concentrations in culled chicks were the greatest, whereas infertile eggs had the lowest values among all hatchery byproducts. The standardized total tract digestibility of phosphorus in infertile eggs was greater than that in unhatched eggs, culled chicks, and the mixture. Further experiments are warranted to determine the influence of dietary hatchery byproducts on the growth performance of nursery pigs.

## Figures and Tables

**Table 1:** Ingredients and chemical compositions of the experimental diets (as-fed basis) in Experiment 1.

Items	Diet				
	Basal	Infertile Eggs	Unhatched Eggs	Culled Chicks	Mixture ^1^
Ingredient, %					
Ground corn	86.70	66.01	66.01	65.67	66.28
Whey powder	10.00	7.61	7.61	7.57	7.64
Infertile eggs	-	25.00	-	-	-
Unhatched eggs	-	-	25.00	-	-
Culled chicks	-	-	-	25.00	-
Mixture ^1^	-	-	-	-	25.00
Ground limestone	1.73	-	-	0.87	-
Monosodium phosphate	0.82	0.63	0.63	0.13	0.33
Sodium chloride	0.40	0.40	0.40	0.40	0.40
Vitamin premix ^2^	0.10	0.10	0.10	0.10	0.10
Mineral premix ^3^	0.25	0.25	0.25	0.25	0.25
Analyzed composition					
Dry matter, %	90.4	90.9	92.1	92.0	91.9
Gross energy, kcal/kg	3850	3971	4006	4369	4274
Crude protein, %	9.5	15.7	16.4	23.7	20.7

^1^ The mixture contained 20% dried infertile eggs, 20% dried unhatched eggs, and 60% dried culled chicks. ^2^ Provided the following quantities per kilogram of complete diet: vitamin A, 20,000 IU; vitamin D_3_, 4000 IU; vitamin E, 180 IU; vitamin K_3_, 2.5 mg; thiamin, 3.5 mg; riboflavin, 8.0 mg; pyridoxine, 5.0 mg; vitamin B_12_, 0.05 mg; pantothenic acid, 25 mg; folic acid, 2.5 mg; niacin, 42 mg; biotin, 0.2 mg; and ethoxyquin, 1.5 mg. ^3^ Provided the following quantities per kilogram of complete diet: Co, 0.5 mg as cobalt sulfate; Cu, 75 mg as copper sulfate, 12.5 mg as copper-methionine; Fe, 125 mg as iron sulfate; I, 1.0 mg as calcium iodate; Mn, 75 mg as manganese sulfate; Se, 0.25 mg as sodium selenite; and Zn, 50 mg as zinc sulfate and 10 mg as zinc-methionine.

**Table 2:** Ingredients and chemical compositions of the experimental diets (as-fed basis) in Experiment 2.

Items	Diet				
	Infertile Eggs	Unhatched Eggs	Culled Chicks	Mixture ^1^	P-Free
Ingredient, %					
Corn starch	45.25	46.25	53.56	50.25	52.02
Infertile egg	25.00	-	-	-	-
Unhatched egg	-	25.00	-	-	-
Culled chick	-	-	25.00	-	-
Mixture	-	-	-	25.00	-
Sucrose	20.00	20.00	20.00	20.00	20.00
Gelatin	9.00	8.00	-	4.00	15.00
Cellulose	-	-	-	-	4.00
Soybean oil	-	-	-	-	3.00
Amino acid mixture ^2^	-	-	-	-	3.02
Ground limestone	-	-	0.69	-	1.71
Potassium carbonate	-	-	-	-	0.40
Magnesium oxide	-	-	-	-	0.10
Sodium chloride	0.40	0.40	0.40	0.40	0.40
Vitamin premix ^3^	0.10	0.10	0.10	0.10	0.10
Mineral premix ^4^	0.25	0.25	0.25	0.25	0.25
Analyzed composition					
Dry matter, %	92.3	93.5	94.1	93.3	92.7
Gross energy, kcal/kg	3984	3943	4241	4127	3998
Crude protein, %	17.5	17.6	17.1	18.0	18.6
Calcium, %	2.89	2.87	0.67	1.23	0.57
Phosphorus, %	0.19	0.20	0.34	0.28	0.01

^1^ The mixture contained 20% dried infertile eggs, 20% dried unhatched eggs, and 60% dried culled chicks. ^2^ Amino acid mixture contained 0.88% l-lysine HCl, 0.23% dl-methionine, 0.19% l-tryptophan, 0.32% l-histidine, 0.48% l-valine, and 0.45% l-isoleucine. ^3^ Provided the following quantities per kilogram of complete diet: vitamin A, 20,000 IU; vitamin D_3_, 4000 IU; vitamin E, 180 IU; vitamin K_3_, 2.5 mg; thiamin, 3.5 mg; riboflavin, 8.0 mg; pyridoxine, 5.0 mg; vitamin B_12_, 0.05 mg; pantothenic acid, 25 mg; folic acid, 2.5 mg; niacin, 42 mg; biotin, 0.2 mg; and ethoxyquin, 1.5 mg. ^4^ Provided the following quantities per kilogram of complete diet: Co, 0.5 mg as cobalt sulfate; Cu, 75 mg as copper sulfate and 12.5 mg as copper-methionine; Fe, 125 mg as iron sulfate; I, 1.0 mg as calcium iodate; Mn, 75 mg as manganese sulfate; Se, 0.25 mg as sodium selenite; and Zn, 50 mg as zinc sulfate and 10 mg as zinc-methionine.

**Table 3:** Analyzed composition of hatchery byproducts (as-is basis).

Items	Infertile Eggs	Unhatched Eggs	Culled Chicks	Mixture ^1^
Dry matter, %	91.1	97.3	98.3	96.5
Gross energy, kcal/kg	4206	4313	5884	5192
Crude protein, %	32.2	37.7	67.5	57.4
Ether extract, %	22.9	21.6	20.8	21.4
Ash, %	31.9	35.6	8.7	16.8
Calcium, %	12.0	12.9	1.8	6.1
Phosphorus, %	0.56	0.65	1.11	0.94

^1^ The mixture contained 20% dried infertile eggs, 20% dried unhatched eggs, and 60% dried culled chicks.

**Table 4:** Energy digestibility and metabolizability in pigs fed experimental diets (as-fed basis) in Experiment 1 ^1,2^.

Items	Diet					SEM	*p*-Value
	Basal	Infertile Eggs	Unhatched Eggs	Culled Chicks	Mixture ^3^		
Diet intake, kg/d	0.63	0.64	0.66	0.65	0.64	0.07	0.858
GE intake, kcal/d	2453 ^b^	2558 ^ab^	2656 ^ab^	2842 ^a^	2750 ^ab^	283	0.011
Dry feces output, kg/d	0.07 ^b^	0.11 ^a^	0.11 ^a^	0.09 ^b^	0.09 ^ab^	0.01	<0.001
GE in dry feces, kcal/kg	4197 ^b^	4195 ^b^	3061 ^c^	4817 ^a^	4480 ^ab^	104	<0.001
Fecal GE output, kcal/d	296 ^c^	472 ^a^	352 ^bc^	417 ^ab^	416 ^ab^	52	<0.001
ATTD of GE, %	87.4 ^a^	81.9 ^b^	87.2 ^a^	86.0 ^a^	84.9 ^a^	0.7	<0.001
DE in diet, kcal/kg	3366 ^d^	3253 ^e^	3495 ^c^	3758 ^a^	3630 ^b^	28	<0.001
Urine output, kg/d	1.01 ^b^	1.60 ^a^	1.44 ^ab^	1.19 ^ab^	1.37 ^ab^	0.28	<0.001
GE in urine, kcal/kg	52 ^b^	39 ^b^	44 ^b^	81 ^a^	61 ^ab^	8	<0.001
Urinary GE output, kcal/d	44 ^c^	55 ^bc^	56 ^bc^	84 ^a^	71 ^ab^	7	<0.001
ME in diet, kcal/kg	3293 ^c^	3169 ^d^	3413 ^b^	3634 ^a^	3517 ^b^	28	<0.001

SEM = standard error of the means; GE = gross energy; ATTD = apparent total tract digestibility; DE = digestible energy; ME = metabolizable energy. ^a–e^ Least squares means within a row without a common superscript differ (*p* < 0.05). ^1^ Each least squares mean represents 8 observations except for the mixture diet (6 observations). ^2^ Diet intake, fecal output, and urine output were based on 5 days of collection. ^3^ The mixture contained 20% dried infertile eggs, 20% dried unhatched eggs, and 60% dried culled chicks.

**Table 5:** Energy values for hatchery byproducts fed to nursery pigs (Experiment 1) ^1^.

Items	Hatchery Byproduct				SEM	*p*-Value
	Infertile Eggs	Unhatched Eggs	Culled Chicks	Mixture ^2^		
As-fed basis						
GE, kcal/kg	4206	4313	5884	5192		
DE, kcal/kg	2759 ^d^	3735 ^c^	4840 ^a^	4224 ^b^	116	<0.001
ME, kcal/kg	2645 ^c^	3625 ^b^	4560 ^a^	3998 ^b^	123	<0.001
Dry matter basis						
GE, kcal/kg	4617	4433	5986	5380		
DE, kcal/kg	3035 ^d^	4057 ^c^	5258 ^a^	4598 ^b^	126	<0.001
ME, kcal/kg	2909 ^c^	3938 ^b^	4954 ^a^	4352 ^b^	134	<0.001
Ratio						
DE:GE	0.66 ^b^	0.87 ^a^	0.82 ^a^	0.81 ^a^	0.02	<0.001
ME:GE	0.63 ^b^	0.84 ^a^	0.78 ^a^	0.77 ^a^	0.03	<0.001
ME:DE	0.96 ^ab^	0.97 ^a^	0.94 ^b^	0.95 ^b^	0.01	<0.001

SEM = standard error of the means; GE = gross energy; DE = digestible energy; ME = metabolizable energy. ^a–d^ Least squares means within a row without a common superscript differ (*p* < 0.05). ^1^ Each least squares mean represents 8 observations except for the mixture (6 observations). ^2^ The mixture contained 20% dried infertile eggs, 20% dried unhatched eggs, and 60% dried culled chicks.

**Table 6:** Digestibility of dry matter (DM) and phosphorus (P) in hatchery byproducts fed to nursery pigs (Experiment 2) ^1^.

Items	Diet				SEM	*p*-Value
	Infertile Eggs	Unhatched Eggs	Culled Chicks	Mixture ^2^		
No. of observations	7	6	7	6		
Feed intake, g/d						
Total feed intake	517	540	603	551	71	0.627
DM intake	481	507	570	517	67	0.554
P intake	0.98 ^b^	1.10 ^b^	2.01	1.60 ^a^	0.16	<0.001
Fecal output						
Total dry feces, g/d	53	55	52	37	11	0.279
P in feces, %	0.76 ^c^	1.26 ^b^	2.32 ^a^	2.34 ^a^	0.16	<0.001
DM fecal output, g/d	52	54	50	35	11	0.242
P output, g/d	0.37 ^b^	0.61 ^b^	1.13 ^a^	1.01 ^a^	0.14	<0.001
Digestibility, %						
ATTD of DM	89.8	90.6	91.8	92.4	1.1	0.066
ATTD of P	64.0 ^a^	44.7 ^b^	43.8 ^b^	35.5 ^b^	4.2	<0.001
STTD ^3^ of P	81.7 ^a^	61.6 ^b^	53.9 ^b^	47.4 ^b^	4.2	<0.001

SEM = standard error of the means; ATTD = apparent total tract digestibility; STTD = standardized total tract digestibility. ^a–c^ Least squares means within a row without a common superscript differ (*p* < 0.05). ^1^ Diet intake and fecal output were based on 5 d of collection. ^2^ The mixture contained 20% dried infertile eggs, 20% dried unhatched eggs, and 60% dried culled chicks. ^3^ Values for STTD were calculated by correcting ATTD values for basal endogenous losses of P. Basal endogenous losses of P were determined in pigs fed the P-free diet at 361 ± 95 mg/kg of DM intake.
